# Role of ACEI/ARBs on antidepressant treatment response assessed through biomarker levels and gene expression analysis in persons with depression: A prospective observational study

**DOI:** 10.1192/j.eurpsy.2026.10585

**Published:** 2026-07-31

**Authors:** S. C. Sarangi, M. Arora, S. Singh, B. N. Patra, A. Sharma

**Affiliations:** 1 Pharmacology; 2 Psychiatry; 3 Biochemistry, All India Institute of medical Sciences, New Delhi, India

## Abstract

**Introduction:**

Depression often shows suboptimal outcomes with antidepressants, with ~66% response and ~37% remission after 6 weeks of treatment (MDD-1 India study). Renin–angiotensin–aldosterone system (RAAS) has been implicated in mood regulation through its effects on neuroplasticity, inflammation, and stress. While ACE inhibitors (ACEIs) and angiotensin receptor blockers (ARBs) are widely prescribed for cardiovascular disease, their influence on antidepressant response and RAAS-related mechanisms remains underexplored.

**Objectives:**

To evaluate influence of ACEI/ARBs on antidepressant treatment response and correlate them with RAAS and depression related biomarkers.

**Methods:**

This prospective observational study included patients with depression who were going to receive antidepressants for 6 weeks. They were divided into two groups: a. AD group: only antidepressants, b. AD+ACEI/ARBs group: antidepressants with ongoing ACEI/ARBs treatment for ≥ 3 months. Depression severity was assessed using Hamilton Depression Rating Scale (HDRS-17) at baseline and 6 weeks. Serum biomarkers (ACE, Ang II, Ang 1–7, renin, BDNF, cortisol) were quantified by ELISA, and gene expression (ACE, AT1R, AT2R, MasR) was analysed in peripheral blood by qRT-PCR. Safety of medications was evaluated through routine laboratory tests.

**Results:**

This study included 186 patients with depression (AD group: n=102; AD+ACEI/ARB group: n=64) and 20 healthy controls. Reduction in HDRS-17 score was greater within the AD+ACEI/ARB group (22.19 ± 7.15 to 10.61 ± 4.00) compared with AD group (21.97 ± 7.54 to 13.24 ± 6.50; p=0.001). Responder rate (≥ 50% reduction in HDRS score from baseline) was higher in AD+ACEI/ARBs (p=0.001) compared to AD group. At baseline AD+ACEI/ARB group had significantly lower serum ACE (p=0.022) compared to AD group. At 6 weeks of follow-up there was significant reduction in Ang II level (p = 0.002) in AD+ACEI/ARBs group compared to AD group. At baseline gene expression analysis showed upregulation of MasR (p = 0.022) and downregulation of ACE (p = 0.049) in the AD+ACEI/ARB group (with ≥ 3 months ACEI/ARB treatment) vs AD group. However, at follow-up there was no significant change among AD vs AD+ACEI/ARBs group in serum Ang 1–7, renin, BDNF, cortisol levels, and gene expression of AT1 and AT2 receptors.

**Conclusions:**

Adjunctive ACEI/ARB therapy improves antidepressant treatment response, which may be ascribed to the modification of RAAS-related biomarkers. This was reflected through reduction in serum ACE and Ang II levels, downregulation of ACE and upregulation of MasR expression.

**Disclosure of Interest:**

None Declared

